# Recent Strategies and Applications for l-Asparaginase Confinement

**DOI:** 10.3390/molecules25245827

**Published:** 2020-12-10

**Authors:** João C. F. Nunes, Raquel O. Cristóvão, Mara G. Freire, Valéria C. Santos-Ebinuma, Joaquim L. Faria, Cláudia G. Silva, Ana P. M. Tavares

**Affiliations:** 1 Laboratory of Separation and Reaction Engineering-Laboratory of Catalysis and Materials (LSRE-LCM), Department of Chemical Engineering, Faculty of Engineering, University of Porto, Rua do Dr. Roberto Frias, 4200-465 Porto, Portugal; jcfn@ua.pt (J.C.F.N.); roc@fe.up.pt (R.O.C.); jlfaria@fe.up.pt (J.L.F.); 2 Department of Chemistry, CICECO-Aveiro Institute of Materials, University of Aveiro, 3810-193 Aveiro, Portugal; maragfreire@ua.pt; 3 School of Pharmaceutical Sciences, Universidade Estadual Paulista-UNESP, Araraquara 14800-903, Brazil; valeria.ebinuma@unesp.br

**Keywords:** l-asparaginase, confinement strategies, nanomaterials, therapeutic agents, acrylamide mitigation, biosensors

## Abstract

l-asparaginase (ASNase, EC 3.5.1.1) is an aminohydrolase enzyme with important uses in the therapeutic/pharmaceutical and food industries. Its main applications are as an anticancer drug, mostly for acute lymphoblastic leukaemia (ALL) treatment, and in acrylamide reduction when starch-rich foods are cooked at temperatures above 100 °C. Its use as a biosensor for asparagine in both industries has also been reported. However, there are certain challenges associated with ASNase applications. Depending on the ASNase source, the major challenges of its pharmaceutical application are the hypersensitivity reactions that it causes in ALL patients and its short half-life and fast plasma clearance in the blood system by native proteases. In addition, ASNase is generally unstable and it is a thermolabile enzyme, which also hinders its application in the food sector. These drawbacks have been overcome by the ASNase confinement in different (nano)materials through distinct techniques, such as physical adsorption, covalent attachment and entrapment. Overall, this review describes the most recent strategies reported for ASNase confinement in numerous (nano)materials, highlighting its improved properties, especially specificity, half-life enhancement and thermal and operational stability improvement, allowing its reuse, increased proteolysis resistance and immunogenicity elimination. The most recent applications of confined ASNase in nanomaterials are reviewed for the first time, simultaneously providing prospects in the described fields of application.

## 1. Introduction

l-asparaginase (ASNase, EC 3.5.1.1) is an amidohydrolase enzyme that catalyses the l-asparagine conversion to l-aspartic acid and ammonia. This enzyme has an important role in the pharmaceutical and food industries [1,2].

ASNase is used in clinical applications of lymphoproliferative disorders due to its anticarcinogenic potential [3,4]. ASNase tumour-inhibitory properties were described for the first time in 1953 by Kidd [5,6], who reported rapid and almost total tumour regression when treating lymphoma-bearing mice with guinea pig serum. In fact, in 1922, Clementi [7] reported the presence of ASNase in the blood serum of a guinea pig. Therefore, the inhibitory action in sick mice was later ascribed to the ASNase activity [8]. Despite its relevant therapeutic application, ASNase must be used with special care since several aspects still require further studies. ASNase causes severe adverse reactions (depending on its source); the major limitation of this upfront biological treatment is the high number of hypersensitivity reactions (reported in 30–70% of patients after ASNase administration from *Escherichia coli*) [9,10].

On the other hand, it is known that native proteases present in the blood system can break down the ASNase molecule and, due to its non-human origin, it has a recognised rapid plasma clearance [11,12]. All of these aspects, together with the fact that enzymes usually have a short half-life (*t_1/2_* = 1.2 days), make the therapeutic application of ASNase challenging. Therefore, there is an urgent requirement to develop strategies to overcome the current drawbacks of ASNase, particularly considering its safety and pharmacokinetic characteristics.

In addition to the ASNase application in the pharmaceutical field, it also has application in the food industry, namely to reduce the acrylamide formation, a carcinogenic compound in heat-processed food products [13]. The pre-treatment of starchy foods with ASNase, before heating, converts l-asparagine to aspartic acid, preventing the acrylamide formation by the Maillard reaction between l-asparagine and carbonyl compounds at high temperatures [13,14]. In 2003, Zyzak et al. [15] reported the ASNase application for acrylamide reduction in a potato matrix. This observation led to the inclusion of monographs on ASNase from *Aspergillus oryzae* and *Aspergillus niger* in World Health Organization (WHO) food additives series in 2008 (59th series) [16] and 2009 (60th series) [17], respectively. However, as the enzyme action could be affected by food composition [13], the ideal ASNase to be used in the food industry must be stable throughout the food processing and proteolysis and, once consumed, it should not cause allergic or toxic reactions [18].

The manufacture of ASNase-based biosensors to detect and/or quantify l-asparagine levels is also considered a promising technology in both clinical and food industries, as it is a more simple, straightforward and specific method compared to spectroscopic techniques [19]. These biosensors’ mechanism of action is based on the measurement of the ASNase activity. The ammonium ions generated during the asparagine hydrolysis lead to a pH variation and subsequent change of colour and absorption wavelength [20].

Besides the restrictive factors discussed above, the use of ASNase in its free form is challenging due to its unstable nature and limitation to a single use. Thus, the improvement of ASNase enzymatic and therapeutic properties has been achieved by introducing chemical modifications and physical integration within several supports. These techniques, if properly designed, can improve the enzymes stability and allow their reuse, also contributing to the reduction of operation costs [21,22,23,24]. Due to enzymes protection (enhanced activity and stability [25,26]) and expanded catalytic half-life [27], confined ASNase can find improved applications in a wide range of areas, namely as sustained or continuous-release delivery systems, as biosensors in clinical diagnosis, as biocatalysts in the food industry, among other [24]. Nevertheless, as the enzymes confinement on support materials could result in several enzyme modifications, the changes in the enzyme structure and activity should be thoroughly studied and evaluated according to the target application [28]. Therefore, the choice of the support material and the confinement procedure, are aspects of maximum importance.

In this review, we describe the recent developments on ASNase confinement strategies based on the latest research advances (since 2018) and their respective applications, not reported in the existing reviews [29,30].

## 2. l-Asparaginase

ASNase can be produced from a wide variety of natural sources, namely microorganisms (bacteria, yeast, filamentous fungi, algae), plants and vertebrates; however, microorganisms are the preferred source for ASNase production in large scale for clinical and industrial applications [31]. Numerous microorganisms are known to be valuable sources of ASNase, including *Aspergillus tamarii* [32], *Aerobacter* spp., *Bacillus* spp., *Photobacterium* spp., *Serratia* spp. and *Xanthomonas* spp. [33], *Pseudomonas aeruginosa* [34], *Proteus vulgaris* [35], *Streptomyces griseus* [36] and *Vibrio succinogenes* [37]; the research to find new ones is still in progress [38]. Nevertheless, commercial ASNase currently used as a therapeutic is purified solely from genetically modified *E. coli* [39,40] or *Dickeya dadantii* (formerly *Erwinia chrysanthemi*) [41] due to their enhanced l-asparagine specificity (essential amino acid for most tumour lymphoblastic cells). Furthermore, since glutamine is able to recover asparagine-deprived cells through asparagine regeneration via a transamidation chemical reaction, successful anti-leukemic ASNase activity might require glutamine reduction in addition to asparagine depletion [42,43,44]. Therefore, ASNase from *Erwinia carotovora* has also emerged as a potential therapeutic enzyme due to its increased glutaminase activity, which may cause fewer side effects when used as an anticancer medicine [42,43,44,45].

In 1967, two ASNase isozymes with different properties were discovered in *E. coli*, namely type I (EcA I) and type II (EcA II) [46]. Type I ASNase is a homodimeric cytosolic constitutive enzyme, while type II ASNase, normally assuming a homotetrameric configuration, is located in the enzyme periplasm, being secreted only as a response to exposure to low nitrogen concentrations [47]. Even though both isozymes show enzymatic activity for l-asparagine and l-glutamine, the main difference between them is the specificity for l-asparagine [40]. EcA II is known to have anti-tumour activity due to the higher specific affinity for l-asparagine (EcA I *K_m_* (Michaelis–Menten constant) = 3.5 mM and EcA II *K_m_* = 10–15 µM), being, consequently, the one used for medical applications [48].

Different researchers have extensively studied ASNase in order to clarify its molecular structure. All type II ASNase from bacteria are tetramers with 222 symmetry and 140–150 kDa [49]. However, depending on the enzyme source, monomeric, dimeric or hexameric forms also are present [19]. The tetramer is composed of identical subunits denominated by A, B, C or D, bound mainly by non-covalent interactions. Each monomer consists of about 330 amino acid residues with 14 α-strands and 8 β-helices organised in two domains—a larger one, the *N*-terminal domain, and a smaller one, the C-terminal domain—linked by approximately 20 residues. The enzyme active site located between the N- and C-terminal domains of two adjacent monomers contains the catalytic nucleophile Thr15 common to all ASNase [49]. 

**Figure 1:**
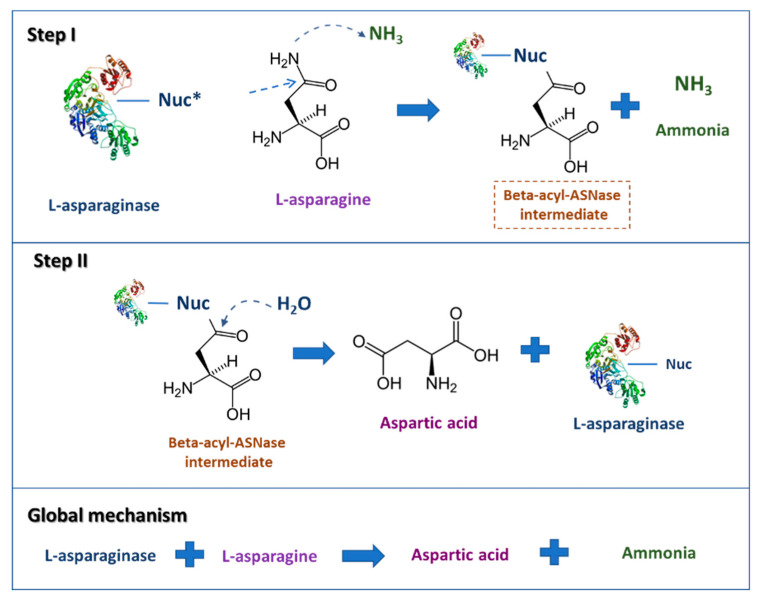
Scheme describing the l-asparaginase reaction mechanisms. * Nuc: nucleophilic residue (adapted from Hill et al. [51]).

The l-asparagine hydrolysis by ASNase occurs in two main steps (see Figure 1). The first step involves the enzyme nucleophilic residue activation by NH_2_, a powerful base, and the subsequent attack on the l-asparagine amide carbon atom, generating the beta-acyl-enzyme intermediate; the second one comprises the nucleophile activation by a water molecule, attacking the ester carbon successively, providing l-aspartic acid and liberating ammonia [47,50].

### Commercial ASNase

Currently, there are few type II ASNase commercially available that have been produced industrially for medical applications (detailed in Table 1): (i) native ASNase from *E. coli* (Elspar^®^ from Ovation Pharmaceuticals, Illinois, IL, USA [52]; Leukanase^®^ from Sanofi-aventis, New South Wales, Australia; Kidrolase^®^ from EUSA Pharma, SAS, Lyon, France [53], etc.); (ii) PEGylated ASNase from recombinant *E. coli*, pegaspargase (Oncaspar^®^ from Enzon Pharmaceuticals, Florida, FL, USA) [54]; (iii) native ASNase, but as a recombinant form, being produced in *E. coli* and *E. chrysanthemi* as host cells (Spectrila^®^ from Medac Gesellschaft, Wedel, Germany [55] and Erwinase^®^ (from *Erwinia chrysanthemi*) from EUSA Pharma, SAS, Lyon, France [56], respectively).

Elspar^®^ was the first ASNase to be available on the market and to be approved (1978) by the U.S. Food and Drug Administration (FDA) for use as a component of a multi-agent chemotherapeutic regimen for the treatment of patients with acute lymphoblastic leukaemia (ALL). In 1994, Oncaspar^®^ received the same approval by FDA, but only for patients with hypersensitivity to native Elspar^®^. It was only in 2006 that it was approved as part of the first-line therapy for any ALL patient [57]. In November 2011, FDA approved Erwinase^®^, indicating its use as a component of a multi-agent chemotherapeutic regimen for ALL patients treatment who have developed hypersensitivity to either Elspar^®^ or Oncaspar^®^ [58]. Finally, in January 2016, the European Commission granted a marketing authorisation valid throughout the European Union for Spectrila^®^ (from *E. coli*). However, all these ASNase products are associated with several noteworthy toxicities and should be used with care because of the possibility of severe reactions, including anaphylaxis and sudden death [59].

Commercially approved ASNases to be used in food industries (detailed in Table 1) comprise the fungal ones from *A. oryzae* (Acrylaway^®^ from Novozymes A/S, Bagsvaerd, Denmark) and *A. niger* (PreventASe^TM^ from DSM, Heerlen, The Netherlands) [60,61]. The US government attributed the status of “generally recognised as safe” (GRAS) to both ASNases and, in 2007 and 2008, they also received a favourable evaluation as a food additive by the Joint FAO/WHO Expert Committee [62].

## 3. Types of ASNase Confinement

Confinement of enzymes allows their continuous use while they are physically or chemically confined or localised in a certain defined area of the support, maintaining their structural integrity and exhibiting better catalytic activity [63,64,65]. Therefore, the choice of the support and enzyme confinement technique should take into account the nature of the enzyme, namely its biochemical and kinetic properties, the nature and type of the support and the purpose of application [66,67,68,69,70].

A high number of ASNase confinement possibilities have been recently developed, which may be grouped into three main approaches: (i) physical adsorption; (ii) covalent attachment; (iii) entrapment. In the next sections, recent developments of each type of ASNase confinement on (nano)materials are overviewed and discussed, emphasising the confinement yield (*η_i_*), ASNase stability improvement, specific pH range, half-lives, enzyme structure, thermal stability (TS), storage stability (SS), operational stability (OS), ASNase cytotoxicity in different cell lines in vitro cytotoxicity (IVtC) and IC_50_, in vivo results and substrate affinity. The ASNase confinement works published since 2018, detailing the used support and confinement technique, as well as the enzyme improved properties, are displayed in Table 2.

### 3.1. ASNase Confinement by Physical Adsorption

**Figure 2:**
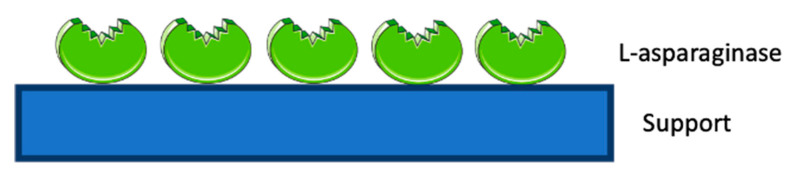
ASNase confinement by physical adsorption.

ASNase confinement by adsorption over nanomaterials includes the physical attachment of the enzyme via non-covalent bonds, namely dispersive interactions, hydrogen bonding and Coulombic interactions (Figure 2). This confinement technique comprises low associated costs, and it may allow support regeneration, improved enzyme performance and easy enzyme reload [66,68,93,94,95].

Different types of (nano)materials such as inorganic, organic, magnetic and hybrid materials have been reported in the literature as supports for ASNase confinement. Over the last twenty years, carbon-based materials have been successfully applied in enzyme confinement due to their porous structure and distinct porous sizes, several surface contact sites, high surface area and adsorption capacity, abundance of functional groups and small release of fine particulate matter [96,97,98,99]. In particular, graphene oxide (GO) offers high potential in several fields, specifically for biotechnological and biomedical applications, mainly due to its large specific surface area, and to the possibility of introducing various oxygen surface groups, namely epoxy, hydroxyl, carboxylic and carbonyl, which provide attachment sites to several biological molecules, such as enzymes [100,101,102]. In 2018, Monajati et al. [71] published the first article about physical and covalent confinement (the last one being described in Section 3.2) of ASNase on GO [30,71]. The physical confinement of ASNase onto aspartic-acid-functionalised graphene oxide nanosheets led to the recovery of 25.1% of its free activity following 24 h at 60 °C (TS), exhibiting 29% of enzymatic activity after 8 cycles of reaction at 60 °C (OS) [71].

Carbon nanotubes (CNTs), another carbon allotrope, are known to be among the most promising nanomaterials for biomedical applications due to their exceptional mechanical, electrical and optical properties [103] and to their ability to be filled with different compounds, including drugs [104]. However, the biomedical application of CNTs raises some questions related to safety and toxicity. CNTs can effectively penetrate the organism; the interaction between them and organs or cells may have consequences of varying severity or even fatal problems [105]. In addition, as CNTs are not well-defined structures, presenting different morphologies, purities, structures and scales, depending on the preparation/purification procedures, determining the involved interactions is difficult and unpredictable. Cui et al. [106] compared the effect of different CNTs on several target cells and reinforced the need for further toxicity studies to better understand this topic. Several authors [107,108,109] defended the idea that specific functionalization methods are able to significantly reduce CNTs toxicity, denoting a promising progress towards CNTs biomedical application. Haroun et al. [72] successfully confined ASNase from *Aspergillus versicolor* onto multi-walled carbon nanotubes (MWCNTs) through physical adsorption, displaying a *η_i_* of 54.4%. Furthermore, confined ASNase kept complete enzymatic activity (100%) after 30 min incubation at 45 °C (TS) and exhibited a higher half-life (1155 min) than free ASNase (173.25 min) at 50 °C, despite displaying a lower substrate affinity (*K_m_* 0.045 M) than free enzyme (*K_m_* 0.02 M) [72]. The confinement of a commercial ASNase from *Escherichia coli* onto MWCNTs by physical adsorption was also evaluated by Cristóvão et al. [73], who attained promising results with a *η_i_* of 100% after 45 min of contact time and a relative recovered activity above 90%. Despite the lower substrate affinity demonstrated by the confined ASNase (*K_m_* 0.109 M) in relation to its free form (*K_m_* 0.047 M), the enzyme confinement led to reaching a higher maximum reaction rate (0.029 mM min^−1^) when compared to the free enzyme (0.019 mM min^−1^). In both articles, the *K_m_* of the confined enzyme was higher than that of the free enzyme. It is known that as the smaller the *K_m_* value, the greater the affinity of the enzyme for the substrate. In this way, both enzymes after confinement showed less affinity for the substrate. It is possible that the adsorption of the enzyme on the MWCNT reduced the number of sites available for binding with the substrate, which generated the lowest values of *K_m_* with the enzyme confined, meaning that the confinement reduced the affinity of the enzyme for substrate. The different values of *K_m_* between the enzymes produced by *A. versicolor* and *E. coli* are due to the structures of the enzymes, which may be different.

Tarhan et al. [74] confined 77.2% (*η_i_*) of total ASNase onto maltose-functionalised magnetic core/shell Fe_3_O_4_@Au nanoparticles (NPs) while increasing the enzyme acid–base tolerance and thermal stability. The bioconjugate kept 90% of its initial activity following 3 h of incubation at 55 °C (TS) and sustained 64% of its activity after 28 days at 25 °C (SS), besides presenting 50% of the initial activity after 13 cycles of reaction (OS) [74]. Confined ASNase *K_m_* (1.59 ± 0.21 mM) is inferior to free ASNase *K_m_* (2.95 ± 0.29 mM), displaying the confined ASNase affinity enhancement for asparagine [74]. 

From our perspective, despite the promising results obtained with MWCNTS, this support is still at an early stage regarding the use for ASNase confinement and further studies are needed to improve its application. Among the described works, maltose-functionalised magnetic core/shell Fe_3_O_4_@Au NPs [74] seem to be the most promising nanomaterial for ASNase confinement by physical adsorption due to the highest *η_i_* displayed (77.2%), along with the high TS (90% of activity after 3 h of incubation at 55 °C) and SS (64% of activity following 28 days at 25 °C), ability to keep half of its initial enzymatic activity after 13 cycles of reaction (OS) and enhanced confined ASNase affinity. The combination of (nano)materials with magnetic properties allows for an easy separation process [22,110,111], providing a good option for the ASNase confinement.

### 3.2. ASNase Confinement by Covalent Attachment

**Figure 3:**
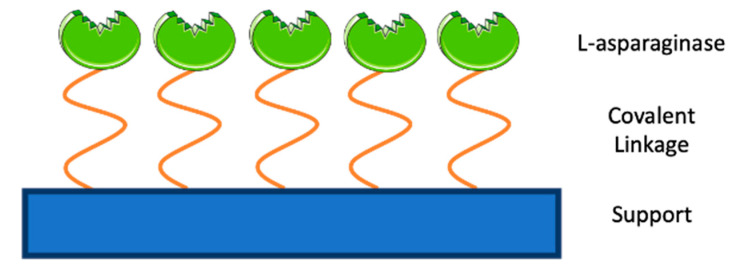
ASNase confinement by covalent attachment.

The covalent confinement (Figure 3) of an enzyme is characterised by the establishment of an irreversible chemical bond between the enzyme functional groups and the support material [112,113]. The stability of the chemical bond is defined by the enzyme binding direction, achieving maximum activity levels when the active centre amino acids are not involved in the support bonding [66,95]. The support linkage is established either via reactive functional groups already present in the support or through support modification to produce activated groups [66,95,114,115].

Silica has been broadly used as an inert and stable support for enzyme confinement due to its tuneable physicochemical characteristics, such as tailorable pore diameters, which may vary from microporous (<2 nm), mesoporous (2–50 nm) or macroporous (>50 nm) silicas, depending on the confined enzyme dimension (3 to 6 nm) [116,117,118]. Furthermore, easily synthesised and low-cost silica NPs present high stability and high surface-to-volume ratio, allowing high levels of enzyme confinement and activity enhancements [75,116,119]. Many enzymes, such as oxido-reductases, hydrolases and isomerases, have been encapsulated within silica NPs [116]. To the extent of our knowledge, Golestaneh and Varshosaz [75], in 2018, published the first report about covalent ASNase confinement onto silica NPs through two distinctive cross-linking agents, namely 1-ethyl-3-(3-dimethylaminopropyl)carbodiimide hydrochloride (EDC) and glutaraldehyde. The optimal pH range for native ASNase and ASNase covalently confined onto silica NPs with EDC was 6.5–7.5, while the range for ASNase covalently confined using glutaraldehyde was 5–8.5. Following 1 h of trypsin digestion (pH 7, 50 international units (IU) trypsin, 37 °C), ASNase covalently confined onto silica NPs with EDC kept 80% of its enzymatic activity, while when using glutaraldehyde as cross-linker it retained 72% of its initial activity (TS). Moreover, stability half-lives of the bioconjugated ASNase were higher than the ones of native ASNase in both phosphate buffer and plasma, besides showing no significant differences between them. Furthermore, Golestaneh and Varshosaz [75] reported an improved ASNase structural stability and affinity to asparagine, reflected in the *K_m_* value decrease from 3.2 ± 0.1 mM in native ASNase to 2.3 ± 0.1 mM and 2.6 ± 0.1 mM in the ASNase covalently confined using EDC and glutaraldehyde, respectively [75]. 

Gold NPs (AuNPs) have distinct characteristics due to their size (<100 nm) and structure that allow their use as supports for enzymes confinement [120,121,122,123,124,125,126], namely: distinctive optical, physical, chemical plus magnetic properties; high surface area; biocompatibility for small biomolecules conjugation; non-cytotoxicity; ability to penetrate deep into tissues or cells; track intracellular trafficking and localisation. Baskar et al. [76] reported the confinement of ASNase from *Aspergillus terreus* onto AuNPs, resulting in an 18.4-fold increase in the protein concentration (0.332 mg mL^−1^) and in a 1.4-fold increase in the specific activity of ASNase (364 U mg^−1^) when comparing with crude asparaginase (0.018 mg mL^−1^ and 252.05 U mg^−1^, respectively) [76].

Metal oxide NPs have arisen as a versatile support for enzyme confinement due to their improved electrical, mechanical, optical, physical and chemical properties, namely nanosize, large specific surface area, low toxicity, susceptibility to modification with different surface functional groups through covalent bonds and biocompatible environment, which aid enhancing ASNase stability and reusability [23,77,127]. Agrawal and Kango [77] reported the covalent confinement of ASNase (from *E. coli*) onto functionalised aluminium oxide NPs (AONP) and titanium oxide NPs (TONP), which were activated via glutaraldehyde, maintaining after 23 days at 37 °C more than 40% and 35% of enzymatic activity (SS), besides keeping an average ASNase activity of 91.8% and 95.1% during nine consecutive cycles of enzyme reaction using AONP and TONP (OS), respectively. AONP-ASNase showed better affinity (*K_m_* 1.9 μM) towards its substrate than free ASNase (*K_m_* 2.9 μM) [77]. Agrawal et al. [78] reported an ASNase (from *E. coli*) *η_i_* of 85% onto aluminium oxide pellets (AlOPs) by covalent attachment using glutaraldehyde as cross-linker, keeping an ASNase activity of 72.97% after 30 days at 4°C (SS). The bioconjugate revealed improved activity and stability at several temperatures and pH values, in addition to enhanced operational stability, being reused for up to nine cycles and keeping an average ASNase activity of 83% (OS). AlOPs also displayed better affinity (*K_m_* 5.39 μM) towards the substrate than free ASNase (*K_m_* 12.8 μM) [78].

Monajati et al. [71] reported a 100% covalent efficiency (*η_i_*) of ASNase confinement over aspartic acid functionalised graphene oxide nanosheets, keeping 40.6% of enzymatic activity following 24 h at 60 °C (TS), and exhibiting 42% of ASNase activity after eight cycles of enzyme reaction at 60 °C (OS) [71].

Ates et al. [79] reported an ASNase (from *E. coli*) *η_i_* of 73.2% onto magnetic Fe_3_O_4_-chitosan NPs, displaying more than 60% of enzymatic activity at 70 °C (TS), and 50% and 48% of ASNase activity after 28 days at 4 °C and room temperature (SS), respectively. Moreover, the confined ASNase kept 60.5% of its initial enzymatic activity after 16 cycles (OS) [79]. Ulu et al. [80] reported an ASNase (from *E. coli*) *η_i_* of 98% after confinement onto epoxy-functionalised Fe_3_O_4_@MCM-41 magnetic NPs, synthesised via co-precipitation of Fe_3_O_4_ core–shell magnetic NPs later coated with MCM-41 silica, retaining: more than 92% of its original activity after 3 h at 55 °C (TS); 54% and 26% of initial activity after 30 days of storage at 4 °C and 25 °C (SS), respectively; and 56.3% of enzymatic activity after 12 consecutive cycles of reaction (OS). Ulu et al. [81] reported an ASNase (from *E. coli*) *η_i_* of 63% onto chloro-modified magnetic Fe_3_O_4_@MCM-41 core-shell NPs, retaining: 69.7% of initial ASNase activity levels after 180 min at 55 °C (TS); 47% and 32.5% of enzymatic activity after 28 days at 4 °C and 25 °C (SS), respectively; and 42.2% of original ASNase activity after 18 cycles of reaction (OS). Furthermore, immobilised ASNase kept more than 85% of its activity in a wide pH range (7.0–9.0). Recently, Orhan and Uygun [82] reported the successful covalent confinement of ASNase onto 117.5 nm averaged sized magnetic poly(HEMA-GMA) NPs, keeping 50% of the initial ASNase activity after 10 h (TS). In addition, it retained 30% of the original enzymatic activity after 40 days (SS) and 85% of ASNase activity after eight successive cycles of reaction (OS). Confined ASNase maintained 74.74% of its original enzymatic activity in artificial serum samples, making these results promising for the future development of in vivo tests [82]. Alam et al. [83] achieved a maximum ASNase (from *B. aryabhattai*) *η_i_* of 62% onto aminopropyl-triethoxysilane (APTES)-modified magnetic NPs, increasing the confined enzyme half-life at 70 °C by almost 3.3 times (TS). The authors also reported an enhancement of the enzyme operational stability through reuse during five cycles with activity losses of less than 10% (OS). The catalytic efficiency (*V_max_*/*K_m_*) of confined ASNase preparations was higher than the *V_max_*/*K_m_* of the free enzyme, confirming that ASNase displayed better affinity towards asparagine [83].

Currently, polymeric nanobiocomposites are applied in several biomedical engineering fields due to their mechanical and biological features, namely their size and biocompatibility, which allow them to function as excellent drug carriers [128]. Thus, through a simple co-precipitation method, followed by ASNase (from *A. terreus*) covalent confinement using glutaraldehyde, Baskar et al. [84] successfully synthesised 60–90 nm cerium selenium ASNase nanobiocomposites. Baskar and Sree [85] prepared a biodegradable 40–80 nm β-cyclodextrin-ASNase nanobiocomposite and Baskar and Sree [86] developed a biodegradable and non-toxic 74.1–80 nm β-cyclodextrin-gelatin-ASNase nanobiocomposite.

As far as we are concerned, within the presented works about ASNase confinement by covalent attachment, the most promising (nano)materials were AONP and TONP [77], presenting an enhanced OS (average ASNase activity of more than 91% during nine consecutive cycles of reaction) and SS (more than 35% of enzymatic activity following 23 days at 37 °C); and magnetic Fe_3_O_4_-chitosan NPs [79], displaying a high OS (60.5% of its initial enzymatic activity following 16 cycles) and SS (almost half of its enzymatic activity following 28 days at room temperature).

### 3.3. ASNase Confinement by Entrapment

**Figure 4:**
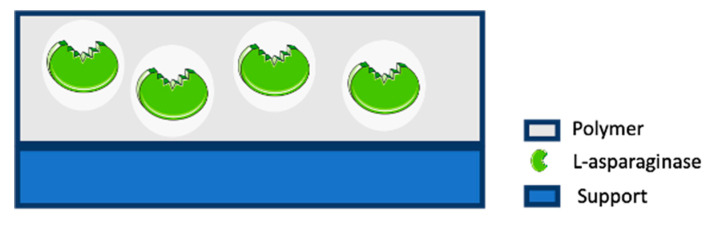
ASNase confinement by entrapment.

Enzyme entrapment comprises enzyme trapping within the framework of a membrane or 3-D polymer support of high-molecular weight compounds, allowing enzyme preservation while substrate diffusion can occur (Figure 4) [129,130,131].

**Figure 5:**
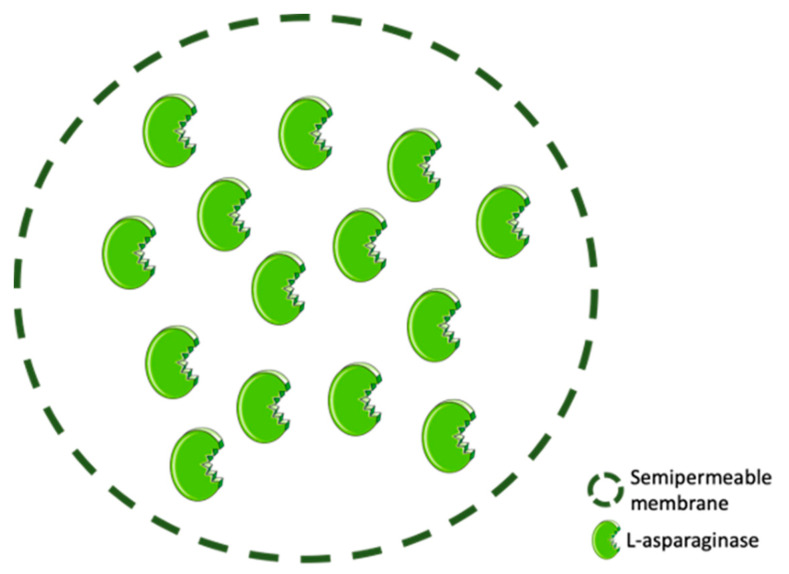
ASNase confinement by microcapsule-type entrapment.

This confinement technique can be divided into lattice-type entrapment, in which the enzyme is trapped by a natural or a cross-linked water-insoluble polymer, namely polyvinyl alcohol or polyacrylamide, and microcapsule-type entrapment, wherein the enzyme is surrounded by a semi-permeable polymer membrane, whose production demands exceptionally well-controlled settings (Figure 5) [130,132,133]. Despite becoming space-restricted, the enzymes remain free in movement, while small substrates can freely cross the semi-permeable membrane [132,133]. 

In recent years, calcium alginate has stood out as a confinement support material due to its physicochemical features in gel form, essential in the entrapped biologically active material reactions outcome [133].

Baky and Baroty [87] successfully entrapped ASNase from *Spirulina maxima* onto natural polymers, within which agar cake beads displayed the highest ASNase *η_i_* and confined enzymatic activity [87].

Since alginate possesses biocompatibility, low toxicity, biodegradability and superior gelling properties, this polyanionic copolymer of 1–4 linked β-d-mannuronic acid and α-l-glucuronic acid extracted from brown algae or bacteria, whose gel is mainly formed by ionic gelation, is frequently applied for cells and enzyme confinement [134,135,136,137,138,139]. Ashok and Devarai [88] produced ASNase from *Rhizopus microsporus* IBBL-2 through microencapsulation into Ca-alginate beads, reaching after 48 h an enzymatic activity of 17.68 U mL^−1^ (TS). 

Poly(lactic-co-glycolic acid) (PLGA) is a polymer approved by the Food and Drug Administration (FDA) and European Medicines Agency (EMA) for drug delivery due to its valuable features, such as biocompatibility, biodegradability, well-described formulations, flexible production methods, degradation drug protection, sustained release, surface characteristics modification and potential to target NPs to certain organs or cells [89,140]. Brito et al. [89] developed an ASNase encapsulation method into PLGA NPs, which were produced by ultrasonic cavitation, a double emulsification technique, exhibiting more than 80% of ASNase loading capacity (*η_i_*). The detected encapsulated ASNase activity levels (265 ± 6 U mg^−1^) were higher than free ASNase activity (213 ± 5 U mg^−1^). Furthermore, encapsulated ASNase release was slower than the in vitro free ASNase release from dialysis bags, since, within 7 and 14 days, 56% and 60% of enzyme was released from the PLGA NPs, whereas following the same time intervals, free ASNase was released for more than 61% and 66%, respectively [89].

Tinoco et al. [90] successfully established a novel method for ASNase encapsulation through emulsification via high-pressure homogenisation using BSA and Pol 407 as core NPs elements (BSA/ASN/Pol_407_ NPs). The leading systems with 15%, 20% and 25% of ASNase whose size was between 61.66 and 73.68 nm and the polydispersity index (PdI) inferior to 0.23, kept the original ASNase activity, while at times even increasing its enzymatic activity after 4 months at 4 °C (SS). Moreover, in vivo toxicity results using the zebrafish embryotoxicity (ZET) protocol proved the safety of BSA/ASN25%/Pol_407_ NPs since ASNase (7 μg mL^−1^) entrapment allowed the increase of zebrafish survival, probably due to its ability to retain ammonia. However, further studies are needed to characterise the NPs’ antileukemic activity in vitro and in vivo using cells and animal models [90].

Possarle et al. [91] managed to insert carbon nanotubes (CNT) in Langmuir–Blodgett (LB) films of stearic acid (SA), which work as supports for ASNase confinement. The LB technique enables the preparation of ultrathin layers, whose bioinspired support provided by the lipid at air–water interfaces is useful for confinement of enzymes since it allows them to maintain its secondary and tertiary structures [141,142]. After 30 days, the SA-ASNase-CNT monolayer and SA-ASNase monolayer kept 85% and 78% of confined ASNase activity, respectively, while the free ASNase only conserved 14% of its original activity (SS). Therefore, the SA-ASNase-CNT monolayer provided suitable ASNase accommodation, helping the analyte access the ASNase catalytic site while preserving its secondary enzymatic structure [91].

Ulu et al. [92], in 2019, published the first work about ASNase entrapment using calcium-alginate/multi-walled carbon nanotube hybrid beads (Ca-ALG/MWCNT-COOH), reaching an ASNase loading yield of 97% (*η_i_*) using 2 mm beads, 187.5 U of ASNase, 0.2 M of CaCl_2_ and 0.5% of alginate (ALG). While free ASNase suffers an irreversible thermal denaturation over 55 °C, entrapped ASNase in Ca-ALG/MWCNT-COOH/LA held 11.13% of the primary enzyme activity at 65 °C due to confined ASNase conformational flexibility limitation (TS). Furthermore, confined ASNase kept 81.2% of its initial enzymatic activity during 4 weeks of incubation at 30 °C (SS). After 14 cycles of reaction, confined ASNase preserved 36.4% of its original enzymatic activity (OS). Confined ASNase *K_m_* value diminished from 0.42 to 0.33 mM, displaying a higher specific affinity for asparagine [92]. 

From our point of view, the most promising (nano)materials presented for ASNase confinement by entrapment was BSA/ASN25%/Pol_407_ NPs [90] displaying features such as small size (62.80 ± 1.41 nm) and homogeneous population (PdI: 0.13 ± 0.010) suitable for an intravenous application, along with an enhanced SS (more or 100% of initial ASNase activity following 4 months at 4 °C) and in vivo safety.

## 4. Applications of Confined ASNase

As described above, several organic, inorganic, hybrid and composite (nano)materials have been investigated for ASNase confinement [99]. Due to the improved properties of confined ASNase, applications in the pharmaceutical and food industries, and as asparagine biosensors, have been reported [143], being summarised in Table 3. The three main applications are more detailed in the following subsections. 

### 4.1. Therapeutic Applications

**Figure 6:**
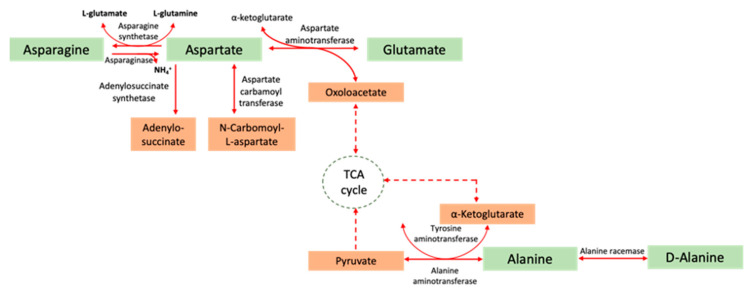
Schematic representation of the asparagine, aspartate and alanine metabolism. (Adapted from Marchese et al. [154]).

Taking into account its antileukemic features, type II ASNase has been applied in the treatment of lymphoproliferative disorders and lymphomas, namely ALL, T-cell lymphomas, subtypes of myeloid leukaemias and NK tumours [3,6,42,43,44]. Furthermore, due to its glutaminase activity, ovarian carcinomas and further solid tumours have also been projected as ASNase additional targets [44]. In fact, in vitro ASNase sensitivity was exhibited for soft tissue sarcoma [145], β-catenin mutated hepatocellular carcinoma [146], hepatocellular carcinoma with low asparagine synthetase expression [147] and gastric adenocarcinoma [148,149]. ASNase can deplete asparagine, an essential amino acid to tumour cells. More specifically, healthy cells synthesise l-asparagine through transaminase enzyme, which converts oxaloacetate into an intermediate aspartate that subsequently transfers an amino group from glutamate to oxaloacetate generating α-ketoglutarate and aspartate, which is transformed into asparagine through asparagine synthase or glutamine-dependent asparagine synthetase via an ATP-dependent reaction which takes advantage of the amido-*N* of l-glutamine in order to form the amido group of asparagine (Figure 6) [19,150,151].

While previously Chan et al. [152] reported that only asparagine-synthetase-positive cancer types need ASNase glutaminase activity, more recently the same author [153] showed that ASNase glutaminase activity is essential for long-lasting, single-agent anticancer in vivo activity against not only asparagine synthetase-positive, but also asparagine-synthetase-negative cancer types. However, additional studies are still required in order to completely figure out the role of cellular glutamine levels regarding ASNase sensitivity, besides glutaminase’s role in ALL evolution [151].

**Figure 7:**
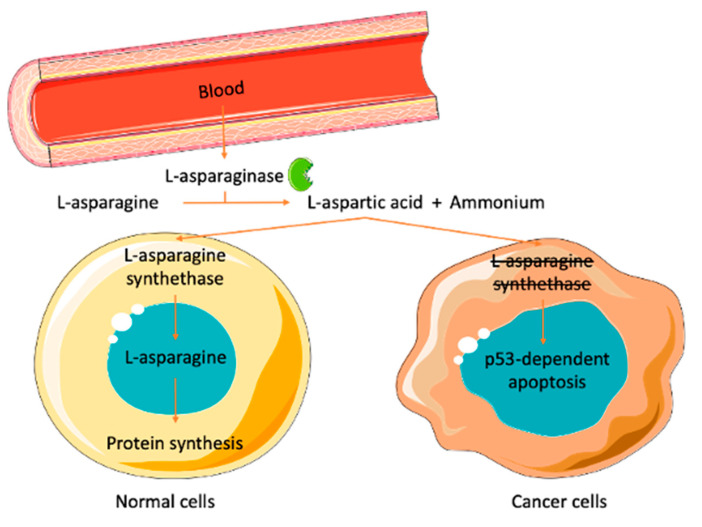
Schematic representation of the antitumoral outcome of l-asparaginase.

In contrast to healthy cells, which express l-asparagine synthetase, cancer cells, mostly of lymphoid origin, rely on exogenous l-asparagine supply from blood serum for their metabolic needs such as quick and malignant growth, spread and survival, since they are auxotrophs for l-asparagine [50,155]. Therefore, asparagine hydrolysation by ASNase from blood serum leads to p53-dependent apoptosis of cancer cells, while healthy cells remain unaffected (Figure 7) [50,155,156].

Despite the ASNase therapeutic potential, more investigation and developments are needed for improving its safety and pharmacokinetic features, since hypersensitivity can lead to anaphylaxis, pain, edema, urticaria, erythema, rash and pruritis. At the same time, immune inactivation can also occur; toxicities are caused either by (i) immunologic sensitisation to a foreign protein or due to (ii) protein synthesis inhibition [9,155,157,158,159], along with the enzyme short half-life (*t_1/2_* = 1.2 days) and fast plasma clearance by native proteases in the blood system [11,12]. ASNase confinement is considered an important approach to overcome these obstacles since it allows higher action time and drug effect, besides lower immune response due to the protection against native proteases, increased ASNase half-life and stability in contrast with free ASNase [30,143]. In fact, Baran et al. [160] showed that the injection of ASNase confined into poly(3-hydroxybutyrate-co-3-hydroxyvalerate) nanocapsules to mice led to longer in vivo confined ASNase circulation lifetime, displaying no side effects and anaphylaxis symptoms, while free ASNase led to immune responses.

Haroun et al. [72] tested the in vitro cytotoxicity of ASNase confined on MWCNTs against normal fibroblast cell line (BHK-21), displaying a stable state profile after 50 μg mL^−1^. Since ASNase causes adverse effects on the liver and pancreas of many patients, Haroun et al. [72] also performed in vivo tests using biochemical biomarkers, such as alanine aminotransferase (ALT), aspartate aminotransferase (AST), lactate dehydrogenase (LDH), lipase and α-amylase in treated male mice. The ASNase confinement decreased its harmful effect on the measured biomarkers, keeping the biomarkers activity values similar to the control group levels [72].

Recent in vitro screening of confined ASNase in distinct cancer cell lines, namely lung cancer cell line (A549) using AuNPs [76], cerium selenium nanobiocomposite [84] and natural polymers such as agar cake beads [87]; ovarian cancer cell line (A2780) via AuNPs [76]; brain cancer cell lines (U87) and cervical cancer cell lines (HeLa) through β-cyclodextrin-gelatin nanobiocomposite [86]; prostate cancer cell lines (PC3) applying β-cyclodextrin nanobiocomposite [85] and natural polymers [87]; human myeloid leukaemia cell line (U937) with β-cyclodextrin nanobiocomposite [85]; ALL cell line (MOLT-4) using AONP [77]; and hepatocellular carcinoma (Hep-G2) cancer cell lines using natural polymers [87] displayed promising anticancer activity (detailed in Table 4). 

Regarding ASNase from *A. terreus*, while the highest in vitro cytotoxicity levels (84.51%) were obtained with AuNPs against A549 cell line [77], the in vitro cytotoxicity levels of AuNPs and free ASNase against the same cell line were already high (73.68% and 74.88%, respectively), thus the in vitro cytotoxicity levels only increased by 10.83% and 9.63%, respectively. Furthermore, concerning ASNase from the same microorganism, the in vitro cytotoxicity levels of cerium selenium nanobiocomposite [84] using the same ASNase dosage (1000 µg mL^-1^) against the same (A549) cell line was still high (70.84%), although it was lower than in the previously mentioned work. It is important to highlight that the control cerium oxide NPs achieved 35.92% of in vitro cytotoxicity levels using a lower ASNase (100 µg mL^−1^) dosage. 

While β-cyclodextrin-ASNase nanobiocomposite with ASNase from *A. terreus* presented promising results (64.79%; 56.42%) using an ASNase dosage of 1000 µg mL^−1^ against PC3 and U937 cell lines [85], respectively, similar cytotoxicity levels were achieved by β-cyclodextrin (61.43%; 50.5%) and ASNase on solution (62.15%; 45.47%) through similar dosages against the same cell lines. Therefore, from our point of view, the most promising nanomaterial for therapeutic applications was β-cyclodextrin-gelatin-ASNase nanobiocomposite [86] due to presenting high in vitro cytotoxicity levels using the same ASNase dosage (1000 µg mL^−1^), namely 78.23% and 82.74% against HeLa and U87 cell lines, respectively. Nevertheless, confined ASNase from *E. coli* in vitro anticancer levels are still low, such as TONP [78] that displayed only 40% of anticancer activity against MOLT-4 cell lines. 

Thus, further investigation in this field is still required since most works display in vitro anticancer levels below 80% and because, to the best of our knowledge, there is only one work using ASNase entrapment for therapeutic applications, which appears to be a promising method because it allows ASNase preservation while remaining free in movement [130,131,132,133].

### 4.2. Food Applications

Acrylamide is classified by the World Health Organization (WHO) and by the International Agency for Research on Cancer (IARC) as a Group 2A carcinogen (“probably carcinogenic to humans”) [162,163]. In 2005, the Food and Agriculture Organization (FAO) and WHO declared the presence of significant amounts of acrylamide in certain processed food or cooked at high temperatures [164]. Table 5 summarizes the acrylamide levels in food products known as to contain higher acrylamide concentrations and the acrylamide intake by individuals >2 years of age collected by the Food and Drug Administration (FDA) between 2011–2015 [165]. The major foods contributors to acrylamide dietary exposure proved to be breakfast cereals and French fries. Acrylamide is formed by the Maillard reaction occurring between reducing sugars and proteins/amino acids at elevated temperatures [166,167]. Several factors related to food products composition have been shown to influence the acrylamide formation levels and temperatures [168,169]. For example, Tareke et al. [170] reported low acrylamide levels formation (between 5–50 µg/kg) during a controlled heating of protein-rich foods, with lower levels in fish. Higher doses of acrylamide (150–1000 µg/kg) were detected in carbohydrate-rich foods like beet root and potatoes. In addition, no acrylamide content was detected on unheated or boiled foods. One of the main substances involved in the Maillard reaction is asparagine, an amino acid often found in food goods [171]. In 2004, Amrein et al. [172] proposed for the first time the use of an enzyme, ASNase, to reduce the acrylamide formation by asparagine hydrolysis in gingerbread. Since ASNase acts on the main reaction precursor, asparagine, and this is not considered a key contributor to the taste and appearance of processed foods, the desired organoleptic properties are maintained [173]. Several ASNase were already used to reduce the acrylamide dosage in a range of food products [174], like potatoes [15], bread [175,176], French fries [14,177], coffee [178], biscuits, crispbread and sliced potatoes chips [179]. Reductions of acrylamide content up to 99% proved the efficiency of using ASNase in food processing [13,180,181]. In 2008, Pedreschi et al. [177] reported the first use of a commercial ASNase (Acrylaway^®^) for acrylamide mitigation, establishing as optimum operating conditions a temperature of 60 °C and a pH of 7.0. While the use of the commercial enzyme Acrylaway^®^ has been reported by several authors, to date, there is only one publication on the use of PreventASe^TM^ for acrylamide mitigation from a food product [175]. To guarantee the safe use of these commercial enzymes in food manufacture, they are deactivated during the heating process [179]. 

When using enzymes in the food industry, it must be considered that factors such as temperature, pH, time and enzyme–substrate ratio are of great importance. Most ASNase are thermolabile and active in a narrow pH range [182]. For example, Acrylaway^®^ activity decreases significantly at temperatures above 60 °C, and may even be denatured [13]. The contact time between the enzyme and the food goods should also be optimised, as well as the ratio between the enzyme and substrate, trying to determine the minimum amount of enzyme to be used to reduce the process costs [13]. For use in the food industry, improving the ASNase stability over a wide range of temperature and pH, as well as having a high substrate specificity, conversion rate and operational stability to reduce the processing time and costs [183] are the most important questions to be overcome, where enzyme confinement may contribute towards this goal. 

Up to date, few works are available on the use of confined ASNase for asparagine reduction, diminishing, consequently, the acrylamide formation. Recently, Alam et al. [83] explored the *Bacillus aryabhattai* ASNase confinement onto APTES-modified magnetic NPs in order to improve enzymatic stability and activity. After incubation of the bioconjugate in a starch–asparagine food model system, it was clearly revealed a decrease in acrylamide formation by more than 90% within 30 min, since no acrylamide peak was detected by HPLC, proving that the ASNase nanoconjugate has a high potential for application in food processing. The improvement of ASNase stability for acrylamide mitigation in fried potato chips by immobilisation on magnetic NPs was described by Aiswarya and Baskar [144], who reported improved OS without enzymatic activity loss during three reuse cycles and an acrylamide reduction of about 75%. Other recently investigated supports involve aluminium oxide pellets (AlOPs) [78]. The pre-treatment of blanched potato chips with extremely thermostable AlOP-ASNase reduced the acrylamide content by 80.5%, proving its effective benefit to be used as a food additive.

### 4.3. Biosensor Applications

A biosensor is an analytical device used to quantify a chemical substance; it combines a biological entity with a signal detector. The living element, microorganisms, enzymes, antibodies, nucleic acid, organelles, etc., interacts, recognises or binds to the substance under analysis. The detector to measure/quantify the analyte is an appropriate physicochemical, optical, electrochemical, thermometric, piezoelectric or magnetic transducer [184]. Asparagine biosensors can be applied either to monitor asparagine levels in blood serum samples of ALL and lymphosarcoma patients or to detect asparagine levels in various food samples [19,185]. The biosensor mechanism is based on the pH change and consequent colour and absorption shift upon ammonia release during the asparagine hydrolysis [20]. This biosensing method provides a high specific, simple and fast response, allowing an online asparagine detection. However, so far, only a few studies regarding the development of ASNase-based biosensors for asparagine quantification have been reported [20,186,187]. The most recent report was made by Possarle et al. [91], in 2020, who published the first label-free approach using smart LB films based on lipids-CNT-enzyme hybrids as sensitive units in optical devices [91]. The supramolecular structure comprising the SA-CNT-ASNase system exhibited enhanced stability and control over the ASNase molecular architecture and accommodation, directly influencing its catalytic activity [91]. Although only a few works were found in this field, the available results are encouraging to develop biosensors for asparagine in biological fluids or food products.

## 5. Conclusions and Perspectives

In this review article, an overview of the recent strategies for ASNase confinement reported since 2018 is presented and the strategies are discussed. Several (nano)materials and distinct confinement techniques have been investigated, namely physical adsorption, covalent attachment and entrapment, whereas applications are focused on the use of ASNase as a therapeutic, to mitigate the formation of acrylamide in food products and as a biosensor to detect the levels of asparagine in biological samples and food products.

While the ASNase tumour-inhibitory properties were originally discovered in 1953, only in 1978 was the first ASNase (Elspar^®^) approved by FDA for ALL treatment. Throughout the years, other ASNase-based drugs have been approved by FDA to treat ALL; however, toxicity and hypersensitivity have been reported. These drawbacks can be overcome by the ASNase confinement in appropriate materials/supports. Even though, in recent years, numerous works about ASNase confinement in which the enhanced biochemical and pharmacological features of ASNase are reported have been published, more work is needed to fulfil the requirements of regulatory agencies and reach the biopharmaceutical industry. Within the recent reports, the ASNase entrapment into BSA/ASN25%/Pol_407_ NPs displayed the most promising results for an intravenous application, high SS and in vivo safety. The ASNase entrapment into (nano)materials has been reported in the literature; however, there are no commercial solutions of this type currently in the market. We highlight that the entrapment confinement method will be important for real applications since it allows ASNase preservation and free movement.

**Figure 8:**
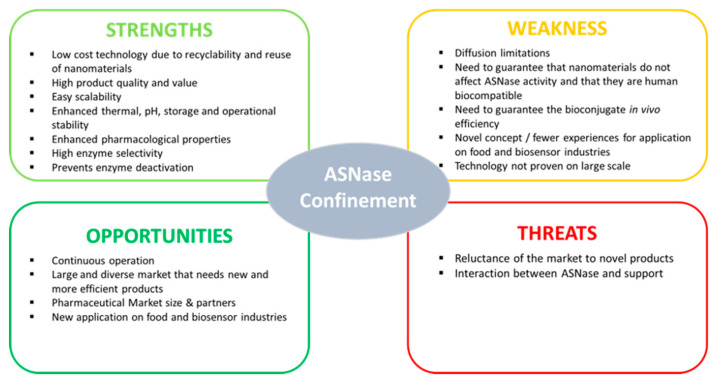
SWOT analysis for the ASNase confinement potential.

In 2004, the potential of using ASNase in the food industry for acrylamide formation reduction was discovered. However, since the majority of ASNases are thermolabile and active in a narrow pH range, ASNase confinement has been recently investigated to improve the enzymatic stability and activity over a broad range of temperature and pH, presenting higher substrate specificity, conversion rate and operational stability in order to lower the processing time and costs. In recent times, confined ASNase also started to emerge in biosensing technology, opening new possibilities up at the industrial level, namely in therapeutic/pharmaceutical and food industries due to its potential to monitor asparagine levels in blood serum samples of ALL and lymphosarcoma patients and to detect asparagine levels in different food samples. The need for a thermostable ASNase to improve the applicable range of temperatures in both the food processing and biosensing industries reveals the importance of the recent findings obtained in the few existing works and the need for further research on the use of confined ASNase in these two industries. To enable assessment of the ASNase confinement potential and identification of areas that need further research, Figure 8 presents a SWOT analysis. We foresee that, in the near future, additional shreds of evidence will confirm the confined ASNase usage at a wider range of industrial application.

## Figures and Tables

**Table 1:** Commercial ASNase for therapeutic/pharmaceutical and food industry applications.

ASNase Application	ASNase Form	Microorganism	ASNase Commercial Name	ASNase Manufacturer
Therapeutic/Pharmaceutical	Native ASNase	*E. coli*	Elspar^®^	Ovation Pharmaceuticals
			Leukanase^®^	Sanofi-aventis
			Kidrolase^®^	EUSA Pharma
	PEGylated ASNase	*E. coli*	Oncaspar^®^	Enzon Pharmaceuticals
	Native recombinant ASNase	*E. coli*	Spectrila^®^	Medac Gesellschaft
		*E. chrysanthemi*	Erwinase^®^	EUSA Pharma
Food Industry	Native ASNase	*A. oryzae*	Acrylaway^®^	Novozymes A/S
		*A. niger*	PreventASe^TM^	DSM

**Table 2:** l-asparaginase confinement by physical adsorption, covalent attachment and entrapment in different supports.

ASNase Confinement Type	Support	ASNase Source	Results	Ref.
Physical Adsorption	Aspartic-acid-functionalised graphene oxide nanosheets	*E. coli* (Medac^®^, Wedel, Germany)	TS ^§^: 24 h (60 °C)–25.1% *	[71]
			OS ^§^: 8 cycles 60 °C–29% *	
	Multi-walled carbon nanotubes (MWCNTs)	*Aspergillus versicolor*	*η_i_*^§^: 54.4%	[72]
			TS: 30 min 45 °C–100% *	
			ASNase half-life: 1155 min (50 °C)	
			*K_m_*^§^: 0.045 M	
			ASNase toxicity: stable after 50 μg mL^−1^ (BHK-21 cell line)	
			In vivo tests: eased ASNase harmful effect (biochemical biomarkers)	
		*E. coli* (Deltaclon S.L., Madrid, Spain)	*η_i_*: 100%	[73]
			Relative recovered activity: >90%	
			*K_m_*: 109 mM	
			*V_max_*^§^: 0.029 mM min^−1^	
	Fe_3_O_4_@Au NPs	*E. coli* Pro-Spec, Ness-Ziona, Israel)	*η_i_*: 77.2 %	[74]
			TS: 3 h 55 °C–90% *	
			SS ^§^: 28 days (25 °C)–64% *	
			OS: 13 cycles–50% *	
			*K_m_*: 1.59 mM	
Covalent attachment	Silica NPs	*E. coli* HAP (Kyowa Hakko Kirin Co, Ltd., Tokyo, Japan)	pH range: 6.5–7.5 ^1 §§^; 5–8.5 ^2 §§^	[75]
			TS (pH 7, 50 IU of trypsin): 1h 37 °C–80% ^1^; 72% ^2^	
			Stability half-lives of the bioconjugated ASNase increase	
			*K_m_*: 2.29 ± 0.10 mM ^1^; 2.57 ± 0.08 mM ^2^	
	AuNPs	*Aspergillus terreus* (CSIR-IMTECH)	Protein concentration: 0.332 mg mL^−1^	[76]
			IVtC ^§^: 84.51% (1000 μg mL^−1^, A549 cell line); 18.51% (100 μg mL^−1^, A2780)	
	AONP ^3^ TONP ^4^	*E. coli* (Sun pharmaceutical Ltd., Mumbai, India)	SS: 23 days 37 °C–> 40 % ^3 §§§^; >35 % ^4 §§§^	[77]
			OS: 9 cycles–91.8% ^3^; 95.1% ^4^	
			*K_m_*: 1.9 μM ^3^	
			IVtC: 61% ^3^ (10 μg mL^−1^, MOLT-4 cell line); 40% ^4^ (10 μg mL^−1^, MOLT-4 cell line)	
	AlOPs	*E. coli* (Sigma-Aldrich, St. Louis, Missouri, USA)	*η_i_*: 85 %	[78]
			SS: 30 days 4 °C–72.97% *	
			OS: 9 cycles–83% *	
			*K_m_*: 5.39 μM	
	Aspartic-acid-functionalised graphene oxide nanosheets	*E. coli* (Medac^®^, Wedel, Germany)	*η_i_*: 100 %	[71]
			TS: 24 h (60 °C)–40.6% *	
			OS: 8 cycles 60 °C–42% *	
	Magnetic Fe_3_O_4_–chitosan NPs	*E. coli* (Pro-Spec)	*η_i_*: 73.2 %	[79]
			TS: 70 °C–>60% *	
			SS: 28 days (4 °C; RT)–50% *; 48% *	
			OS: 16 cycles–60.5% *	
	Epoxy-functionalised Fe_3_O_4_@MCM-41 magnetic NPs	*E. coli* (Sigma-Aldrich, St. Louis, MI, USA)	*η_i_*: 98%	[80]
			TS: 3 h 55 °C–>92% *	
			SS: 30 days (4 °C; 25 °C)–54% *; 26% *	
			OS: 12 cycles–56.3% *	
	Chloro-modified magnetic Fe_3_O_4_@MCM-41 Core–Shell NPs	*E. coli* (Sigma-Aldrich)	*η_i_*: 63 %	[81]
			TS: 3 h 55 °C–69.7% *	
			SS: 28 days (4 °C; 25 °C)–47 % *; 32.5% *	
			OS: 18 cycles–42.2% *	
			pH range (7.0–9.0): >85% *	
	Magnetic poly(HEMA-GMA) NPs	*E. coli* (Sigma-Aldrich)	TS: 10 h–50 % *	[82]
			SS: 40 days–30% *; OS: 8 cycles– 5% *	
			IVtA ^§^: 74.74% *	
	APTES-modified magnetic NPs	*B. aryabhattai*	*η_i_*: 62 %	[83]
			TS: 70 °C –3.3 folds increase	
			OS: 5 cycles–90% *	
			Better substrate affinity S-A ^§^: >90% acrylamide mitigation (30 min)	
	Cerium selenium nanobiocomposite	*Aspergillus terreus* MTCC 1782 (CSIR-IMTECH)	MTT assay ^§^: 70.84% (1000 μg mL^−1^); 48.78% (IC_50_ 125 μg mL^−1^) (A549 cell line)	[84]
	β-cyclodextrin- ASNase nanobiocomposite	*A. terreus* MTCC 1782 (CSIR-IMTECH)	IVtC: 64.79% at 1000 μg mL^−1^ (PC3 cell lines); 56.42% at 1000 μg mL^−1^ (U937 cell lines)	[85]
			IC_50_^§^: 125 μg mL^−1^ (PC3 cell lines); 500 μg mL^−1^ (U937 cell lines)	
	β-cyclodextrin-gelatin-ASNase nanobiocomposite	*A. terreus* MTCC 1782 (CSIR-IMTECH)	IVtC: 78.23% at 1000 μg mL^−1^ (HeLa cell lines); 82.74% at 1000 μg mL^−1^ (U87 cell lines)	[86]
Entrapment	Agar cake beadsAgarose piecesGelatin blocks	*Spirulina maxima*	IC_50_: 22.54 μg mL^−1^ (A549 cell line); 24.65 μg mL^−1^ (Hep-G2 cell line); 56.61 μg mL^−1^ (PC3 cell lines)	[87]
	Ca-alginate beads	*Rhizopus microsporus* IBBL-2	TS: 48 h–17.68 U mL^−1^	[88]
	PLGA NPs	(Changzhou Qianhong BioPharma Co. Ltd., Changzhou, China)	*η_i_*: 80%	[89]
			Encapsulated ASNase activity: 265 ± 6 U mg^−1^	
			Encapsulated ASNase release (7 and 14 days): 56 % and 60%	
	BSA/ASN/Pol_407_ NPs	(Changzhou Qianhong BioPharma Co. Ltd.)	PdI ^§^ > 0.23	[90]
			SS: 4 months 4 °C (systems with 15 %, 20 % and 25 % of ASNase)– ≥ 100 % *	
			ZET protocol ^§^: in vivo safety	
	SA-ASNase-CNT	*E. coli* (Sigma-Aldrich)	SS: 30 days–85% * (monolayer)	[91]
	Ca-ALG/MWCNT-COOH	*E. coli* (Pro-Spec)	*η_i_*: 97%	[92]
			TS: 65 °C–11.13 % *	
			SS: 4 weeks 30 °C–81.2 % *; OS: 14 cycles–36.4% *	
			*K_m_*: 0.33 mM	

* ASNase activity (%). ^§^ Abbreviations: thermal stability (TS); operational stability (OP); confinement yield (*η_i_*); Michaelis-Menten constant (*K_m_*); maximum reaction rate (*V_max_*); storage stability (SS); in vitro cytotoxicity (IVtC); room temperature (RT); in vitro (artificial serum medium) ASNase activity (IVtA); starch-asparagine food model (S-A); 3-(4,5-dimethylthiazol-2-yl)-2,5-diphenyltetrazolium bromide assay (MTT assay); half maximal inhibitory concentration (IC_50_); polydispersity index (PdI); zebrafish embryo toxicity (ZET). ^§§^ Cross-linking agents: ^1^ (1-ethyl-3-(3-dimethylaminopropyl)carbodiimide hydrochloride (EDC)); ^2^ (Glutaraldehyde). ^§§§^ Supports: ^3^ (aluminium oxide nano particles (AONP)); ^4^ (titanium oxide nano particles (TONP)).

**Table 3:** Applications of confined ASNase in pharmaceutical and food industries, and in the development of biosensors.

Applications	Support	ASNase Confinement Type	Ref.
**Therapeutic/Pharmaceutical (Chemotherapeutic Agent)**			
Stable drug support	MWCNTs	Covalent confinement	[72]
Novel effective drug against lung cancer	AuNPs		[76]
Potential anti-lung-cancer drug	Cerium selenium nanobiocomposite		[84]
Potential therapeutic agent for cervical and brain cancer	β-cyclodextrin-gelatin-ASNase nanobiocomposite		[86]
Potential therapeutic agent for prostate cancer and lymphoma	β-cyclodextrin-ASNase nanobiocomposite		[85]
New therapeutic system for drug delivery and anticancer therapy	AONP		[77]
Potential anti-lung-, anti-liver- and anti-prostate-cancer drug	Agar cake beads, agarose pieces and gelatin blocks	Entrapment	[87]
**Food Industry (Acrylamide Mitigation)**			
Efficient biocatalyst for the reduction of acrylamide in S-A food model system	APTES-modified magnetic NPs	Covalent confinement	[83]
Effective in asparagine cleaving for acrylamide mitigation without significant changes in reducing sugar content during frying of potato slices	Nanomagnetic particles		[144]
Acrylamide formation mitigating during commercial processing of starchy foods, namely blanched potato chips	AlOPs		[78]
**Biosensor**			
Sensitive units for asparagine detection in optical devices	SA-ASNase-CNT	Entrapment	[91]

**Table 4:** Confined ASNase cytotoxicity levels for therapeutic applications.

ASNase Confinement Support	ASNase Source	Cell Lines	Confined ASNase Cytotoxicity (%) ([ASNase] (µg mL^−1^) )	NPs Cytotoxicity (%) ([ASNase] (µg mL^−1^))	Free ASNase Cytotoxicity (%) ([ASNase] (µg mL^−1^))	Ref.
AuNPs	*A. terreus*	A549	IVtC *: 84.51 % (1000 µg mL^−1^)	IVtC: 73.68%	IVtC: 74.88%	[76]
		A2780	IC_50_ **: 18.51% (100 µg mL^−1^)	—	—	
Cerium selenium nanobiocomposite	*A. terreus*	A549	IVtC: 70.84% (1000 µg mL^−1^)	IC_50_ (24 h): 35.92% (100 µg mL^−1^)	—	[84,161]
			IC_50_: 48.78% (125 µg mL^−1^)			
β-cyclodextrin-gelatin-ASNase nanobiocomposite	*A. terreus*	HeLa	IVtC: 78.23% (1000 µg mL^−1^)	IC_50_: 51.4% (62.5 µg mL^−1^)	—	[86]
		U87	IVtC: 82.74% (1000 µg mL^−1^)	IVtC: 2.84% (7.8 µg mL^−1^); 57.87% (500 µg mL^−1^)	—	
β-cyclodextrin-ASNase nanobiocomposite	*A. terreus*	PC3	IVtC: 64.79% (1000 µg mL^−1^)	IC_50_: (250 µg mL^−1^)	IVtC: 62.15% (1000 µg mL^−1^)	[85]
				IVtC: 63.04% (1000 µg mL^−1^)		
		U937	IVtC: 56.42% (1000 µg mL^−1^)	IC_50_: (1000 µg mL^−1^)	IVtC: 45.47% (1000 µg mL^−1^)	
				IVtC: 50.5% (1000 µg mL^−1^)		
AONP	*E. coli*	MOLT-4	IVtC: 61% (10 µg mL^−1^)	IVtC: 20% (10 μg mL^−1^)	—	[77]
TONP	*E. coli*	MOLT-4	IVtC: 40% (10 µg mL^−1^)	IVtC: 17% (5 μg mL^−1^)	—	[77]
Natural polymers: agar cake beads	*Spirulina maxima*	A549	IC_50_: (22.54 µg mL^−1^)	—	—	[87]
		Hep-G2	IC_50_: (24.65 µg mL^−1^)	—	—	
		PC3	IC_50_: (56.61 µg mL^−1^)	—	—	

* IVtC: in vitro cytotoxicity; ** IC_50_: half maximal inhibitory concentration.

**Table 5:** Food products ranked by acrylamide levels and respective average intake values collected by FDA between 2011–2015 [165].

Food Product	Acrylamide Level (µg/kg)	Average Acrylamide Intake (µg/kg bw/day)
Cereals	<10–1354	0.050
French Fries and other Potato Foods	<10–1999	0.047
Potato Chips	140–8440	0.038
Cookies and Granola Bars	<10–1796	0.030
Crackers	<10–2110	0.022
Snack Foods	<10–3060	0.019
Coffee	70–1080	0.018
Breads and Bakery Products	<10–102	0.008
